# Antioxidant, anti-adipocyte differentiation, antitumor activity and anthelmintic activities against *Anisakis simplex* and *Hymenolepis nana* of yakuchinone A from *Alpinia oxyphylla*

**DOI:** 10.1186/1472-6882-13-237

**Published:** 2013-09-26

**Authors:** Rong-Jyh Lin, Chuan-Min Yen, Tzung-Han Chou, Feng-Yu Chiang, Guey-Horng Wang, Ya-Ping Tseng, Lin Wang, Ting-Wei Huang, Hui-Chuan Wang, Leong-Perng Chan, Hsiou-Yu Ding, Chia-Hua Liang

**Affiliations:** 1 Department of Parasitology, Faculty of Medicine, Kaohsiung Medical University, Kaohsiung, Taiwan; 2 Department of Chemical and Materials Engineering, National Yunlin University of Science and Technology, Yunlin, Taiwan; 3 Department of Otolaryngology-Head and Neck Surgery, Kaohsiung Medical University Hospital, Kaohsiung Medical University, Kaohsiung, Taiwan; 4 Department of Cosmetic Science, Chia Nan University of Pharmacy and Science, Tainan, Taiwan; 5 Institute of Basic Medical Sciences, National Cheng Kung University, Tainan, Taiwan; 6 Department of Medical Laboratory Science and Biotechnology, Kaohsiung Medical University, Kaohsiung, Taiwan; 7 Department of Public Health, Kaohsiung Medical University, Kaohsiung, Taiwan; 8 Institute of Cosmetic Science, Chia Nan University of Pharmacy and Science, Tainan, Taiwan; 9 Institute of Clinical Medicine, Kaohsiung Medical University, Kaohsiung, Taiwan

**Keywords:** Yakuchinone A, Antioxidant, Adipogenesis, Apoptosis, *Hymenolepis nana*, *Anisakis simplex*

## Abstract

**Background:**

*Alpinia oxyphylla* is a common remedy in traditional Chinese medicine. Yakuchinone A is a major constituent of *A*. *oxyphylla* and exhibits anti-inflammatory, antitumor, antibacterial, and gastric protective activities.

**Methods:**

Antioxidant and antitumor characteristics of yakuchinone A in skin cancer cells as well as novel mechanisms for the inhibition of adipocyte differentiation, cestocidal activities against *Hymenolepis nana* adults, and nematocidal activities against *Anisakis simplex* larvae are investigated.

**Results:**

Yakuchinone A presents the ability of the removal of DPPH·and ABTS^+^ free radicals and inhibition of lipid peroxidation. Yakuchinone A suppresses intracellular lipid accumulation during adipocyte differentiation in 3 T3-L1 cells and the expressions of *leptin* and *peroxisome proliferator-activated receptor* γ (*PPAR*γ). Yakuchinone A induces apoptosis and inhibits cell proliferation in skin cancer cells. The inhibition of cell growth by yakuchinone A is more significant for non-melanoma skin cancer (NMSC) cells than for melanoma (A375 and B16) and noncancerous (HaCaT and BNLCL2) cells. Treatment BCC cells with yakuchinone A shows down-regulation of Bcl-2, up-regulation of Bax, and an increase in cleavage poly (ADP-ribose) polymerase (PARP). This suggests that yakuchinone A induces BCC cells apoptosis through the Bcl-2-mediated signaling pathway. The anthelmintic activities of yakuchinone A for *A. simplex* are better than for *H. nana*.

**Conclusions:**

In this work, yakuchinone A exhibits antioxidative properties, anti-adipocyte differentiation, antitumor activity, and anthelmintic activities against *A. simplex* and *H. nana*.

## Background

Free radicals include superoxide anion (O_2_^-^), hydroxyl (HO·), peroxyl (ROO·), alkoxyl (RO·) and nitric oxide, which are oxygen-centered free radicals occasionally known as reactive oxygen species (ROS). Cellular oxidative damage that is caused primarily by ROS is a well-established general mechanism for cell as well as tissue injury [1,2]. ROS are strongly associated with lipid peroxidation, which leads to the deterioration of the food, and are also involved in a variety of diseases including cellar aging, mutagenesis, carcinogenesis, coronary heart disease, diabetes mellitus, and neurodegeneration [2].

Obesity has become a global health problem due to its association with various metabolic disorders such as type-II diabetes, cardiovascular disease, hypertension, and non-alcoholic fatty liver disease [3,4]. Synthetic anti-obesity drugs have been reported to be costly, and some of them also beset with undesirable side effects. Therefore, developing drugs to directly modulate energy metabolism without affecting the central nervous system has caused substantial attention [4,5].

Natural/herbal compounds including berberine, resveratrol, and curcumin are known to modulate obesity either through increasing energy expenditure or inhibiting adipocyte differentiation [6-8]. Presently the focus is to develop natural compounds as antioxidants that are possibly used to reduce damage caused by oxidative stress, age-dependent diseases, and obesity [9].

*Hymenolepis nana* is a general occasion of cestode infections, and is found worldwide. In human adults, the tapeworm is more of a nuisance than a health problem, but in small children, *H. nana* is dangerous. It is often seen in children in countries with inadequate sanitation and hygiene. *H. nana* infections are typically asymptomatic but heavy infections also cause headaches, anorexia, weakness, abdominal pain, and diarrhea [10]. *H. nana* is the only cestode without any intermediate hosts in its life cycle [11]. *H. nana* infection is typically acquired from eggs in the feces from another infected individual, which are transferred by contaminated food. Eggs hatch in the duodenum, releasing oncospheres that penetrate the mucosa and enter the lymph channels of the villi. Then, oncospheres develop into a cysticercoid, which has a tail and a well formed scolex. About five to six days cysticercoids migrate into the lumen of the small intestine and attach before maturing. Eggs of *H. nana* infect when passed with stool and transfer in contaminated food. Eggs are ingested by an arthropod intermediate host and hatch in the duodenum, releasing oncospheres, and develop into cysticercoid larvae. Upon rupture of the villus, the cysticercoids return to the intestinal lumen, evaginate their scoleces, attach to the intestinal mucosa, and mature into adults that reside in the ileal portion of the small intestine, producing gravid proglottids. The eggs are then passed in stools when released from the proglottids or disintegration of proglottids in the small intestine. An alternate mode of infection consists of internal autoinfection without passing through the external environment. The short life span and rapid course of development also facilitates the spread and ready availability of this worm, but internal autoinfection allows the infection to continue for years [11,12].

*Anisakis simplex* adult worms mature and release eggs from the primary host. The eggs pass from stool into seawater and are embryonated to form *A. simplex* first-stage larvae (AsL1) and subsequently moulted to *A. simplex* second-stage larvae (AsL2). When larvae are ingested by small crustacean first intermediate hosts, the AsL2 matures into *A. simplex* third-stage larvae (AsL3) that are subsequently consumed by second intermediate hosts such as marine fish or squid. The AsL3 migrate into the viscera and peritoneal cavity. The degree of migration into the fish musculature depends on environmental conditions and/or the species of parasite and fish condition [13,14]. AsL3 are repeatedly transferred between fish and fish through the food chain. Therefore, piscivorous fish accumulate large numbers of AsL3 [14]. Finally, the ingestion of infected fish or squid by a marine mammal (i.e. the final host) leads to the development of fourth-stage larvae and then adults. Humans may be accidental hosts by consuming undercooked and/or raw second intermediate hosts that contain AsL3. *A. simplex* rarely develop further within the human gastrointestinal tract, instead, by means of proteolytic enzymes, but they typically embed in the gastric or intestinal mucosa and die or invasion the muscular layers of the stomach and intestine to induce allergic reactions and a variety of abdominal symptoms that are characterized as anisakiasis or anisakidosis [15]. The four main clinical syndromes in humans who experience symptomatic anisakidosis include gastric, intestinal, extra-gastrointestinal, and allergic diseases. Anisakidosis is globally recognized as a public health problem, which is relative to Asia and Europe [16,17]. The prevalence of anisakidosis has increased unusually because of the increasing popularity of Japanese cuisine, such as “sushi” and “sashimi”. The availability of an anthelmintic compound against *A. simplex* has the potential to shorten the clinical course and prevent mechanical invasion that cause from endoscopic procedures. Because few effective studies for anthelmintic drugs and nature compounds against *A. simplex*, the effectiveness of treatment with anthelmintic agents, antibiotics, anticholinergics, and/or corticosteroids against *A. simplex* remains controversial [18].

*Alpinia oxyphylla* is an important traditional Chinese medicinal herb whose fruits are widely used as a tonic, aphrodisiac, anti-salivation, anti-polyuria, and anti-diarrhea [19]. The extracts from *A. oxyphylla* possess neuroprotective activity, anti-tumor, anti-anaphylactic, and inhibition of nitric oxide production [19,20]. Yakuchinone A [1-(4′-hydroxy-3′-methoxyphenyl)-7-phenyl-3-heptanone], a major pungent ingredient derived from *A*. *oxyphylla* exhibits anti-inflammatory, antitumor, antibacterial, antiviral, and gastric protective activities [21]. Yakuchinone A has been reported to be a strong inhibitor of prostaglandin biosynthesis *in vitro*[22]. Moreover, yakuchinone A can act as an anti-tumor promoter as determined by the ability to suppress phorbol ester-induced activation of ornithine decarboxylase (ODC) and inhibits the promotion of papilloma formation in mouse skin [23]. 12-*O*-tetradecanoylphorbol-13-acetate (TPA)-stimulated superoxide generation and tumor necrosis factor-α (TNF-α) or interleukin-1α production in human promyelocytic leukemia (HL-60) cells as well as on DNA binding of activator protein 1 (AP-1) in mouse fibroblast (NIH3T3) cells are also suppressed by yakuchinone A [23,24]. Furthermore, yakuchinone A induces apoptotic death in HL-60 cells account for the antiproliferative activity [23]. However, the biochemical mechanisms underlying the antioxidant, anti-obesity, anti-skin cancer effects of yakuchinone A and its cestocidal effects on *H. nana* and larvicidal effects on *A. simplex* remain unclear. This study confirms the antioxidant and antitumor effects of yakuchinone A and elucidates the novel mechanisms for its inhibition of adipocyte differentiation as well as its anthelmintic activities against *H. nana* and *A. simplex*.

## Methods

### Materials

1,1-Diphenyl-2-picrylhydrazyl (DPPH^•^), 2,2′-azinobis(3-ethylbenzothiazoline-6-sulfonic acid) diammonium salt (ABTS^•+^), 2,5,7,8-tetramethylchroman carboxylic acid (trolox), trichloracetic acid (TCA), 2-thiobarbituric acid (TBA) and 3-isobutyl-1-methylxanthine (IBMX) were purchased form Sigma Chemical Co. (Sigma, St. Louis, MO).

### Extraction and isolation

The “Yizhiren”, *A. oxyphylla*, was supplied from Kwong-Te Co., Kaohsiung, Taiwan and was identified by professor Hang-Ching Lin of the National Defense Medicinal Center, where a voucher specimen was deposited (CNUPS No.970801). The dry powder of *A. oxyphylla* seed (6.0 kg) was extracted with 95% ethanol at room temperature. After removal of the solvent by evaporation, the residue (559.0 g) was dissolved in methanol–water (9.5:0.5) and partitioned with *n*-hexane. The methanol (95%) was removed by evaporation and the residue was then suspended in water and partitioned with ethyl acetate (359.0 g). The ethyl acetate layer was subjected to LH-20 Sephadex and eluted with methanol. Each fraction collected from the column was monitored by thin-layer chromatography and the similar fractions were combined to produce 4 fractions. The fraction 3 was further purified by a silica gel and eluted with *n*-hexane-ethyl acetate (9:1, 7.5:2.5, 1:1, 2.5:7.5), ethyl acetate, ethyl acetate-methanol (9:1), methanol to isolate yakuchinone A (276.1 mg). Their structures were confirmed by NMR and mass spectra analysis.

**Figure 1:**
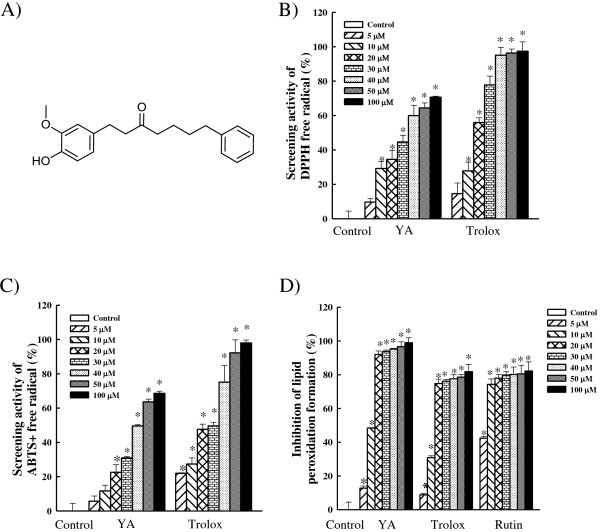
**Antioxidant activity of yakuchinone A. A)** Chemical structure of yakuchinone A from *Alpinia oxyphylla* Miq*.* M.W. = 312. **B)** DPPH · and **C)** ABTS · ^+^ free radical scavenging activities of yakuchinone A and trolox (5, 10, 20, 30, 40, 50, and 100 μM). **D)** Inhibition of lipid peroxidation by yakuchinone A, trolox, and rutin (5, 10, 20, 30, 40, 50, and 100 μM) using liposome as an oxidizable substrate. Data are presented as mean ± SD from three independent experiments; *p < 0.05 indicates significant difference from vehicle-treated cells. Yakuchinone A; YA.

Yakuchinone A: slightly yellow oil; EI/MS *m/z* (rel. int.%): 312(80, [M]^+^), 194 (6), 179 (45), 161 (14), 151 (33), 137 (100), 119 (23); ^1^H-NMR (CDCl_3_, 500 MHz) δ: 1.60 (4H, m, H-5,6), 2.40 (2H, t, *J* =7.0 Hz, H-4), 2.60 (2H, t, *J* =7.0 Hz, H-7), 2.68 (2H, t, *J* =7.6 Hz, H-2), 2.82 (2H, t, *J* =7.6 Hz, H-1), 3.86 (3H, s, OCH_3_), 6.66 (1H, dd, *J* =8.0, 2.0 Hz, H-6’), 6.68 (1H, d, *J* =2.0 Hz, H-2’), 6.83 (1H, d, *J* =2.0 Hz, H-5), 7.15 ~ 7.20 (3H, m, H-3”, 4”, 5”), 7.26 ~ 7.29 (2H, m, H-2”, 6”); ^13^C-NMR (CDCl_3_, 125 MHz) δ: 210.3 (C-3), 146.3 (C-3′), 143.8 (C-4′), 142.1 (C-1″), 133.0 (C-1′), 128.2 (c-2″, 6″), 128.3 (C-3″, 5″), 125.7 (C-4″), 120.7 (C-6′), 114.3 (C-5′), 111.0 (C-2′), 55.8 (OCH_3_), 44.6 (C-2), 42.9 (C-4), 35.7 (C-7), 30.9 (C-6), 29.5 (C-1), 23.3 (C-5). These data were compared with literature values [25]. The chemical structure of yakuchinone A was shown in Figure 1A. The purity of yakuchinone A is 99.2%. The solubility of yakuchinone A was 100 mM in dimethylsulfoxide (DMSO).

### Assay for free radical scavenging ability against DPPH· and ABTS^+^

The radical scavenging activities of yakuchinone A against DPPH · and ABTS · ^+^ radicals were measured by using the method as previously reported [26]. For DPPH · radical scavenging activity analysis, 5, 10, 20, 30, 40, 50, and 100 μM yakuchinone A (10 μl of solution) was mixed with 90 μl of DMSO and 900 μl of ethanolic DPPH · solution (0.1 mM). After incubation in darkness at 25°C for 30 min, the absorbance (*A*) was determined at 517 nm (Hitachi U-2001, Japan). For ABTS^•+^ radical scavenging activity analysis, ABTS · ^+^ was dissolve in water to 7 mM. ABTS · ^+^ radical was produced by reacting ABTS · ^+^ stock solution with 2.45 mM potassium persulfate, and the mixture stood in the dark at room temperature for 12–16 h. The ABTS · ^+^ radical solution was diluted to an absorbance of 0.70 ± 0.02 at 734 nm at 30°C. Each agent (0.1 ml) reacted with 2.9 ml of diluted ABTS · ^+^ radical solution for 20 min at 30°C, and then the absorbance was measured at 734 nm (Hitachi U-2001, Japan). The TEAC (trolox equivalent antioxidant capacity) of the reagent was calculated by comparing their reactivities to the standard antioxidant, trolox. Ethanol or distilled water was used as negative controls. Trolox was used as a standard antioxidant. The scavenging ability of yakuchinone A or trolox in DPPH · and ABTS · ^+^ was calculated using the following equation: radical scavengingability (%) =  (1 - *A*_sample_/*A*_control_) × 100. EC_50_ values were estimated from the percent inhibition versus concentration plot derived from the percentage scavenging activity. This data was shown as mean values ± standard deviation (*n* = 3).

### Determination of antioxidant effect on liposome peroxidation

The effect on liposome peroxidation was assayed by measuring concentrations of thiobarbituric acid reactive substances (TBARS). Liposomes were prepared according to the method of Chou et al. [27]. In brief, the liposomes were obtained by dispersing lipids in demineralized water (1:10). For the assay, 32 μl of suspension of liposomes was incubated together with 11 μl of 10 mM FeSO_4_, 11 μl of 10 mM ascorbic acid and appropriate amounts of different concentrations (5, 10, 20, 30, 40, 50 and 100 μM) of yakuchinone A, trolox and rutin in 1.515 ml of 50 mM Na_2_HPO_4_-NaH_2_PO_4_ buffer, pH 7.4 (2.5 ml final solution) at 37°C for 1 h. Lipid peroxidation was terminated by the reaction of 0.8 ml of 1% TBA and 10% TCA and 106 μl of 0.1 M ethylene diamine-tetraacetic acid disodium salt dehydrate at 100°C for 20 min. After cooling and centrifugation (2600 *g* for 10 min), the malonaldehyde (MDA)-TBA complex was determined by measuring the absorbance (*A*) at 532 nm. A control with DMSO instead of sample was also analyzed and expressed no activity. Trolox and rutin were utilized as standards. The percentage inhibition was calculated using the following equation: Inhinition of lipid peroxidation (%) = (1 - *A*_sample_/*A*_control_) × 100. EC_50_ values were estimated from the percentage inhibition versus concentration plot. This data was shown as mean values ± standard deviation (*n* = 3).

### Cell lines

Human epidermoid carcinoma A431, human oral squamous cell carcinoma SCC25. human skin malignant melanoma A375, mouse melanoma B16, mouse leukemic monocyte macrophage RAW 264.7, mouse normal embryonic liver BNLCL2 cells, and 3 T3-L1 preadipocytes were purchased from the American Type Culture Collection (Rockville, MD). Human basal cell carcinoma BCC and human premalignant keratinocytic HaCaT cells were kindly donated by Prof. Hamm-Ming Sheu (National Cheng Kung University Medical College, Tainan, Taiwan). Cells were cultured in medium supplemented with 10% fetal bovine serum (Hazelton Product, Denver, PA) and 1% penicillin-streptomycin at 37°C in 5% CO_2_ humidified atmosphere; specifically, A431, A375, B16, HaCaT, RAW 264.7, BNLCL2, and 3 T3-L1 cells were maintained in DMEM medium (GIBCO, Grand Island, NY), BCC cells in RPMI medium, and SCC25 in DMEM/F12 medium supplemented with 0.4 μg/ml hydrocortisone (Sigma, St. Louis, MO).

### Adipocyte differentiation

Cultivation of 3 T3-L1 cells and their conversion to adipocytes were carried out according to the method as described previously [28]. To induce differentiation, four day postconfluent 3 T3-L1 preadipocytes were stimulated for 72 h in 10% FBS/DMEM with containing the MDI hormone mixture (0.5 mM IBMX, 1 μM dexamethasone, and 10 μg/ml of insulin) in six-well plates. After four days, the medium was replaced with 10% FBS/DMEM medium containing 10 μg/ml of insulin. The medium was replaced with fresh medium (10% FBS/DMEM, 10 μg/ml of insulin) every two days until analysis on day eight. Yakuchinone A (5 μM) was added during the differentiation process.

### Oil Red O staining

Differentiated 3 T3-L1 cells were stained using the Oil Red O method [29] for adipocyte lipid accumulation. At day eight of differentiation, the cells were washed with PBS and fixed with 10% formaldehyde for 2 h. The fixed cells were washed with 60% isopropanol, and stained with 0.2% Oil Red O for 10 min. The plates were rinsed three times with water and examined under a phase contrast inverted light microscope (Nikon, TE2000-U, Japan). After thorough washing with water and evaporation of excess water, Oil Red O was extracted in isopropyl alcohol and the absorbance was monitored at 520 nm (BioTek, Synergy™2).

### Cell viability

Cells (1 × 10^5^ cells/ml) were plated in 100 μl of 96-well multidishes and treated with a series of concentrations (5, 10, 20, 30, 40, and 50 μM) of yakuchinone A or vehicle control (DMSO) for 72 h. The control groups were treated with DMSO, and the final DMSO concentration did not exceed 0.1%. The cell viability was measured by performing the MTT [3-(4,5-dimethyl-thiazol-2-yl)-2,5-diphenyl-tetrazolium bromide] assay [30]. The IC_50_ values were calculated from the agent concentrations that yielded a cell viability of 50%.

### Cell morphological changes

Cells (1 × 10^5^ cells/ml) were plated in 24-well plates then treated with vehicle control (DMSO) or yakuchinone A (20 μM) for 72 h. Cells in each well were washed once with 1× PBS, and analysis was performed using a phase contrast inverted light microscope (Nikon, TE2000-U, Japan). To assess specific apoptosis, after incubation, cells were washed by PBS and fixed with 4% paraformaldehyde and stained with Hoechst 33342 (0.1 μg/ml) (Sigma) at 37°C for 10 min in the dark. The nuclear morphology changes were viewed under a fluorescent microscope (Nikon, TE2000-U, Japan).

### RNA isolation and reverse transcription-polymerase chain reaction (RT-PCR) analysis

3 T3-L1 cells were treated with vehicle control (DMSO) or yakuchinone A (5 μM) during the differentiation process. BCC cells (1 × 10^5^ cells/ml) were treated with vehicle control (DMSO) or yakuchinone A (20 μM) for 24 and 48 h. Total RNA was prepared from cells using the Trizol reagent (Invitrogen, Carlsbad, CA, USA), and a RT-PCR was conducted using 3 μg of total RNA and the Superscript cDNA Preamplification System (Weiterstadt, Germany) according to the manufacturers’ instructions. The following primers were utilized: right primer 5′-GCT CTA GAC GTG ACA ATC TGT CTG AGG TCT GTC AT-3′ and left primer 5′-CGG CAT CCG TTG TCG GTT TCA CAA ATG CCT TGC AGT G-3′ for PPAR γ (870 bp), right primer 5′-CAT CTG CTG GCC TTC TCC AA-3′ and left primer 5′-ATC CAG GCT CTC TGG CTT CTG-3′ for leptin (71 bp), right primer 5′-AGA TGT CCA GCC AGC TGC ACC TGA C-3′ and left primer 5′-AGA TAG GCA CCC AGG GTG ATG CAA GCT-3′ for bcl-2 (367 bp), right primer 5′-AAG CTG AGC GAG TGT CTC AAG CGC-3′ and left primer 5′-TCC CGC CAC AAA GAT GGT CAC G-3′ for bax (366 bp), and right primer 5′-ACC CAC ACT GTG CCC ATC TA-3′ and left primer 5′-CGG AAC CGC TCA TTG CC-3′ for β-actin (286 bp). The amplified RT-PCR products were analysed in 2% agarose gels, visualized by ethidium bromide staining and photographed under ultraviolet light.

### Western blotting

Cells (1 × 10^5^ cells/ml) were treated with vehicle control (DMSO) or yakuchinone A (20 μM) for 72 h. Then, cells were washed with PBS, and lysed in lysis buffer [50 mM Tris–HCl, pH 7.5, 1% Triton X-100, 5 mM EGTA (ethylene glycol-bis(2-aminoethylether)-*N*,*N*,*N*’,*N*’-tetraacetic acid), 150 mM NaCl and 1 mM phenylmethylsulfonyl fluoride (PMSF)]. After centrifugation (10,000 *g*, 10 min), supernatants were collected. The cell lysates containing 40 μg of solubilized protein were subjected to 12% sodium dodecyl sulfate-polyacrylamide gel electrophoresis (SDS-PAGE) and electrophoretically transferred to nitrocellulose membranes. The membranes were blocked in 5% skim milk. Blots were incubated with the antibodies against Bcl-2, Bax, PARP and β-actin (Santa Cruz, CA). The membranes were incubated with the appropriate secondary antibody conjugated with horseradish peroxidase (Bio-Rad, Hercules, CA). Blotted antibodies were visualized by chemiluminescence method (ECL kit, Amersham).

### Preparation of *H. nana* adult worms

*H. nana* adult worms were obtained from each part of the intestines of wild type mice, purchased from Lin’s farm in Fengshan, Kaohsiung, Taiwan. These parts of the intestine were duodenum, jejunum, ileum, colon and rectum. The *H. nana* had an average length of 5–50 mm and was collected using a needle with a blunt tip, before being placed in Petri dishes with 0.9% NaCl and gentamycin (10 mg/ml). They were then washed several times. The adult worms were individually observed under an inverted microscope, with subsequent discarding of those that exhibited internal or external damage. The adult worms were then identified by their morphological features, divided into groups and placed in 24-well plates contained cultivated media RPMI-1640 plus 20% FBS, pH 7.4, in an atmosphere of 95% O_2_/5% CO_2_, 37°C. These culture conditions have been shown to maximize the development and survival of *H. nana*.

### Assay of cestocidal activity of oscillation and peristalsis test on *H. nana*

The above *H. nana* cultivated media were supplemented with L-glutamine (2 mM), penicillin (100 IU/ml), streptomycin (100 mg/ml) and amphotericin B (0.25 μg/ml), and then the effects of yakuchinone A at concentrations of 10, 50 and 100 μM were tested. The survival and mobility of the adult worm were assessed at 2, 4, 6, 12, 24, 48, and 72 h using a stereomicroscope. They were observed for their spontaneous motility and evoked responses at 2, 4, 6, 12, 24, 48, and 72 h using a stereomicroscope. The oscillation and peristalsis states of adult worms were scored blindly by two investigators. Cestode activity was scored by monitoring both oscillation and peristalsis. Oscillation was scored of movement at scolex and neck for each second for 30 seconds, and then the highest score was 30. Peristalsis was record the contraction real times at scolex and neck. All data were compared with the initial time before the test compounds had been added. Worms death and complete standstill as determined by none any oscillation and peristalsis changes for 30 seconds were identified. The mortality was recorded after ascertaining that the worms neither moved when shaken vigorously nor when dipped in warm medium [31].

### *A. simplex* larvae preparation

The AsL3 were obtained from the muscle and peritoneum of fresh *Trichiurus lepturus*s (largehead hairtail, Atlantic cutlassfish) that were purchased from the fish market of Kaohsiung, Taiwan. The AsL3 had an average length of 20–22 mm, and were collected using a needle with a blunt tip, placed in Petri dishes with 0.9% NaCl and washed several times. The majority of the larvae were encysted, but they quickly became excysted upon washing in NaCl solution. They were individually observed under an inverted microscope, with subsequent discarding of those that exhibited internal or external damage. The larvae were then identified by morphological features, divided into groups and placed in 24-well plates contained cultivated media RPMI-1640 plus 20% FBS, pH 4.0, in an atmosphere of 95% O_2_/5% CO_2_, 37°C. These showed culture conditions demonstrated to provide for the maximum development and survival of *A*[18,32]*.*

### Assay of nematocidal activity on *A. simplex*

The above AsL3 cultivated media were supplemented with L-glutamine (2 mM), penicillin (100 IU/ml), streptomycin (100 mg/ml) and amphotericin B (0.25 μg/ml), and tested of yakuchinone A for 10, 100, and 200 μM. The survival and mobility of the larvae were assessed at 2, 4, 8, 12, 24, 48 and 72 h using a stereomicroscope. Two investigators blindly scored the larvae as dead, with poor mobility or with normal mobility. The percentage losses of spontaneous motion during 3 min periods immediately after incubation and complete standstill were determined by stimulation 4–5 h later (defined as death). The mortality was recorded after ascertaining that the worms neither moved when shaken vigorously nor when dipped in warm medium. The nematocidal activity was modified according to a scoring system that was developed by Kiuchi et al. [33] and Lin et al. [18].

### Statistical analysis

The results are expressed as mean ± standard deviation (SD). Statistical differences were estimated by one-way analysis of variance (ANOVA) followed by Dunnett’s test or the Tukey-Kramer test. A *p* value of 0.05 was regarded as significant. The data were analyzed and the figures plotted using software (SigmaPlot Version 8.0 and SigmaStat Version 2.03, Chicago, IL).

## Results and discussion

### Free radical scavenging activity of yakuchinone A

The DPPH · and ABTS · ^+^ radical has been widely used for assessment of radical scavenging because of the easy and convenient consideration [34]. The soluble free radical DPPH · is well known as a good hydrogen abstractor that yields DPPH-H as a by-product. Thus, the scavenging of DPPH radicals by phenols is effective. The antioxidant activity of yakuchinone A and trolox (a positive control) was measured based on scavenging activities for stable DPPH radical as presented in Figure 1B. With increasing doses from 5 to 100 μM of yakuchinone A and trolox, the values of DPPH · scavenging activity were 9.6%, 29.2%, 34.5%, 44.6%, 60.0%, 64.5%, and 70.7% for yakuchinone A and 14.5%, 27.8%, 55.9%, 77.7%, 95.0%, 96.3%, and 97.4% for trolox, respectively. The EC_50_ values of yakuchinone A for the scavenging of DPPH · radicals were 33.5 (yakuchinone A) and 17.9 μM (trolox). The generation of ABTS · ^+^ involves the direct production of the blue/green ABTS · ^+^ chromophore through the reaction of potassium persulfate and ABTS. The addition of hydrogen-donating antioxidants to the preformed radical reduces it to ABTS [35]. Figure 1C shows the scavenging activity of yakuchinone A towards ABTS · ^+^. As increasing doses of 5, 10, 20, 30, 40, 50, and 100 μM of yakuchinone A and trolox, the values of ABTS · ^+^ scavenging capacity were 5.7%, 11.7%, 22.5%, 31.0%, 49.6%, 63.6%, and 70.6% for yakuchinone A and 21.9%, 27.3%, 47.6%, 49.6%, 75.1%, 92.2%, and 98.0% for trolox, respectively. The EC_50_ values for the scavenging of ABTS · ^+^-radicals were 40.2 (yakuchinone A) and 30.1 μM (trolox). The extent of decolorization as percentage inhibition of the ABTS · ^+^ radical cation was proportional to the concentration of antioxidants and calculated relative to the reactivity of trolox as a standard (TEAC). The TEAC value derived from the dose–response curve for yakuchinone A was 3.4 mM of trolox/g. These results suggest that yakuchinone A exhibits an antioxidant capacity to scavenge DPPH · and ABTS · ^+^ free radicals.

### Potential of yakuchinone A to inhibit lipid peroxidation

The antioxidant action is assessed by inhibiting the damage caused by free radicals and the mechanisms involved in many human diseases such as hepatotoxicities, hepatocarcinogenesis, diabetes, malaria, acute myocardial infarction, and skin cancer to include lipid peroxidation as a main source of membrane damage [9]. Lipid peroxidation in biological systems has been thought to be a toxicological phenomenon that leads to various pathological consequences. MDA formed from lipid peroxidation of unsaturated phospholipid reacts with TBA to produce a pink MDA-TBA adducts. MDA is reactive and active in cross-linking with DNA and proteins and damages liver cells [36]. Phospholipids are believed to be present in high amounts in cell membranes [37]. The phospholipid prepared as a liposome was used to evaluate the effect of yakuchinone A on liposome peroxidation to investigate yakuchinone A in a biological system. Figure 1D presents the inhibition of lipid peroxidation by yakuchinone A (5, 10, 20, 30, 40, 50, and 100 μM) depended on dose. The EC_50_ values of the inhibition of lipid peroxidation efficiency by yakuchinone A, trolox and rutin were 10.3, 14.3 and 6.2 μM, respectively. Although the inhibition of lipid peroxidation activity by yakuchinone A was weaker than by rutin, the inhibition efficiency of yakuchinone A exceeded trolox. The MDA lowering effect of yakuchinone A indicates a protective action against lipid peroxidation of unsaturated phospholipids.

### Inhibition of lipid accumulation by yakuchinone A in 3 T3-L1 adipocytes

**Figure 2:**
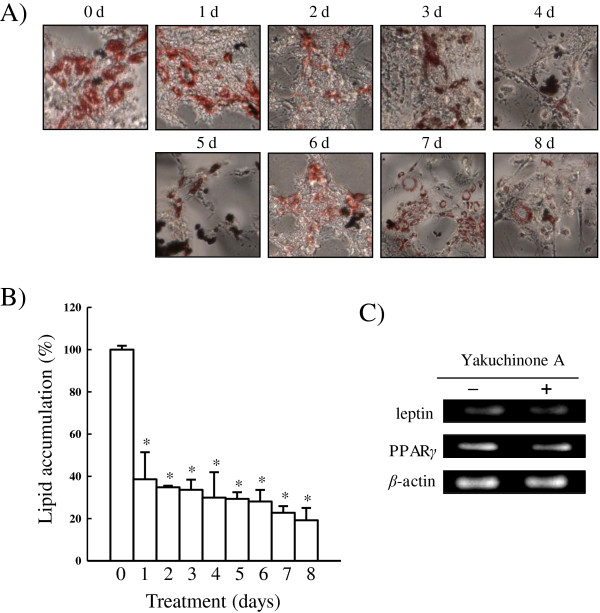
**Yakuchinone A inhibits adipocyte differentiation. A)** Photomicrograph of Oil Red O stained differentiating 3 T3-L1 cells. Cells were treated with yakuchinone A (5 μM) for eight days. Lipid accumulation was measured by Oil Red O staining. **B)** Percentage lipid accumulation was analyzed by quantitative analysis of Oil Red O staining. Data are presented as mean ± SD from three independent experiments; *p < 0.05 indicates significant difference from vehicle-treated cells. **C)** The gene expressions of *leptin*, *PPAR*γ, and β*-actin* were determined by RT-PCR.

Numerous studies show that obesity may induce systemic oxidative stress, and the increase in ROS in adipocytes contributes to deregulated expression of inflammatory cytokines such as tumor necrosis factor-α, which may be an early instigator of the obesity-associated diabetes and cardiovascular disease [37,38]. This work demonstrates that yakuchinone A exhibits anti-oxidation activities, suggesting yakuchinone A has an inhibitory effect on adipogenesis. 3 T3-L1 adipogenic differentiation requires a network of adipogenic markers [3]. We examined the ability of the yakuchinone A to prevent adipogenesis in 3 T3-L1 adipocytes. The amount of accumulated intracellular lipid droplets were compared in differentiated 3 T3-L1 cells after treatment in a MDI mixture and differentiated cells. The amount of intracellular lipid droplets increased in differentiated 3 T3- L1 cells, as shown by the Oil Red O staining (Figure 2A). However, incubation of differentiated cells with low concentration of yakuchinone A (5 μM) decreased MDI-induced lipid accumulation. This result was further supported by quantitative spectrophotometric analysis of cellular neutral lipid content. Figure 2B shows lipid accumulation was significantly inhibited in the presence of 5 μM yakuchinone A. The level of lipid accumulation over eight days was 19.2% of the MDI-treated positive control cells. Adipocytokines are adipocyte-derived hormones, such as leptin and adiponectin, which modulated hepatic and peripheral lipid and glucose metabolism [4]. The amount of leptin secreted in the adipose tissue is positively correlated with the lipid content and adipocyte size [4]. Furthermore, previous research has established that adenosine 5′-phosphate-activated protein kinase (AMPK) and peroxisome proliferator-activated receptor γ (PPARγ) appears to be involved in adipocyte differentiation and maturation. This can be potential drug targets for the treatment of obesity [3]. We evaluated the yakuchinone A-induced changes in the expression of adipose tissue genes associated with adipogenesis through RT-PCR analyses. As shown in Figure 2C, addition of yakuchinone A (5 μM) suppressed the expression of *leptin* and *PPAR*γ significantly as revealed by RT-PCR. These results suggest that yakuchinone A inhibits and adipogenesis due in part to the inhibition of angiogenesis. These events may be mediated, in part, through antioxidative properties of yakuchinone A responsible for inhibition of angiogenesis.

### Effect of yakuchinone A on cell viability and skin cancer cell apoptosis

**Figure 3:**
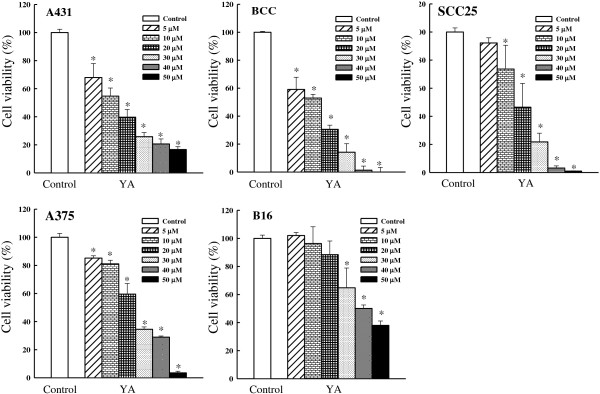
**Effect of yakuchinone A on cell viability in skin cancer cells.** Cell viability of yakuchinone A (5, 10, 20, 30, 40, and 50 μM) to skin cancer (A431, BCC, SCC25, A375, and B16) cells for 72 h, and assessed by MTT assay. Each value is presented as mean ± SD of three individual experiments; *p < 0.05 indicates a significant difference from vehicle control (DMSO)-treated cells. Yakuchinone A; YA.

**Figure 4:**
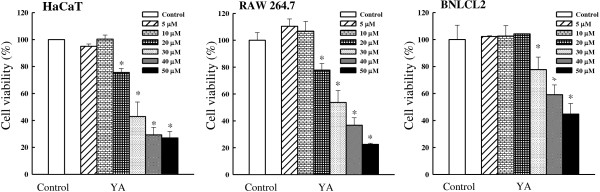
**Effect of yakuchinone A on cell viability in noncancerous cells (HaCaT and BNLCL2) and RAW 264.7 cells.** Cells were treated with yakuchinone A (5, 10, 20, 30, 40, and 50 μM) for 72 h. Cell viability was evaluated with MTT assay. Each value is presented as mean ± SD of three individual experiments; *p < 0.05 indicates a significant difference from vehicle control (DMSO)-treated cells. Yakuchinone A; YA.

Previous report have demonstrated that yakuchinone A exhibits no cytotoxicity against human lung adenocarcinoma A549 cells, human colorectal carcinoma HT-29 cells, and human gastric cancer SGC-7901 cells at a concentration of 10 μg/ml [39], but yakuchinone A induces apoptotic death in HL-60 cells [23]. Nevertheless, cytotoxic effects of yakuchinone A on skin cancer cells remain poorly understood. In this work, the inhibition potential of yakuchinone A on human skin cancer cells (epidermoid carcinoma A431 cells, basal cell carcinoma BCC cells, squamous cell carcinoma SCC25 cells and malignant melanoma A375 cells) and mouse melanoma B16 cells was determined by MTT assay and morphological change. Treatment these cells with yakuchinone A (5, 10, 20, 30, 40, and 50 μM) for 72 h resulted in a dose-dependent significant cell death (Figure 3). The IC_50_ values of yakuchinone A were 13.3, 11.3, 18.7, 23.8, and 40.0 μM for A431, BCC, SCC25, A375, and B16 cells, respectively. Moreover, after 72 h treatment with yakuchinone A (5, 10, 20, 30, 40, and 50 μM), the IC_50_ values of yakuchinone A against noncancerous cells (human premalignant keratinocytic HaCaT cells and mouse embryonic liver BNLCL2 cells) and mouse leukemic monocyte macrophage RAW 264.7 cells were 22.2, 32.2, and 46.4 μM, respectively (Figure 4). Yakuchinone A appeared to have a more potent inhibitory effect on non-melanoma skin cancer (NMSC) cells (A431, BCC, and SCC25) and cell viability than in melanoma cells (A375 and B16), noncancerous cells (HaCaT and BNLCL2), and RAW 264.7 cells. Previous studies have demonstrated that yakuchinone A has a phenolic diarylheptanoid moiety with a carbonyl functional group to suggest that yakuchinone A is anticipated to exhibit potential cancer chemopreventive activities [39]. These experimental data further suggest that yakuchinone A has an antioxidant affect that exhibits less toxic to noncancerous cells and selective cytotoxicity to NMSC cells.

**Figure 5:**
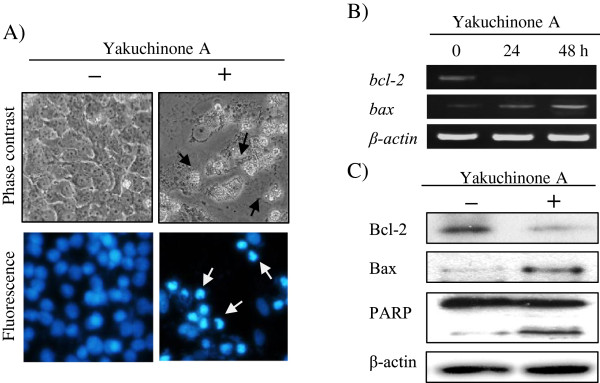
**Expression of Bcl-2 and bax-dependent apoptotic pathway in BCC cells after yakuchinone A treatment. A)** Morphological changes induced by yakuchinone A in BCC cells. BCC cells were treated with yakuchinone A (20 μM) and vehicle control (DMSO) for 72 h, and then the nuclear was stained with Hoechst 33342. Apoptotic cells (arrows) were characterized by cellular shrinkage and rounded cell bodies (phase-contrast-inverted microscopic, 200×). Under a fluorescent microscope, apoptotic cells (arrows) were characterized by marked nuclear condensation, shrinking and fragmentation (200×). **B)** Effect of yakuchinone A on bcl-2 and bax expressions. BCC cells were treated with yakuchinone A (20 μM) and vehicle control (DMSO) for 24 and 48 h, and the *bcl-2*, *bax* and β*-actin* expressions were determined by RT-PCR. **C)** Expressions of Bcl-2, Bax and PARP on cells after yakuchinone A treatment. BCC cells were treated with (+) or without (-) yakuchinone A (20 μM) for 72 h, and the Bcl-2, Bax, PARP and β-actin expression were assessed by Western blotting.

The cell death induction by yakuchinone A was further confirmed by cellular morphological examination. After exposure of 20 μM yakuchinone A to BCC cells at 72 h, distinct cytoplasmic shrinkage, cell bodies became rounded and detached from the surface under phase-contrast-inverted microscopic examination (Figure 5A). Treatment of BCC cells with yakuchinone A showed chromatin condensation and nuclear fragmentation by Hoechst 33342 staining under a fluorescent microscope, indicating apoptosis (Figure 5A). Bcl-2 family members are major apoptosis-regulating proteins [40]. Given that the Bcl-2 family proteins are known mediators of mitochondrial functions, expression levels of anti-apoptotic protein Bcl-2, and pro-apoptotic protein Bax were determined. Bcl-2 expression was time-dependent decreased; whereas, bax was increased and investigated by RT-PCR following the exposure of BCC cells to yakuchinone A (20 μM) for 24 and 48 h (Figure 5B). These experimental results are consistent with the yakuchinone A (20 μM) applied for 72 h by Western blotting (Figure 5C). Cleavage of the poly (ADP-ribose) polymerase (PARP) in BCC cells after yakuchinone A treatment gave further evidence that apoptosis happened because the active form of PARP, a protein associated with DNA repair, is considered as a hallmark of apoptosis. These results suggest that yakuchinone A-induced cell death is mainly due to apoptosis.

### Cestocidal activity against *H. nana*

**Figure 6:**
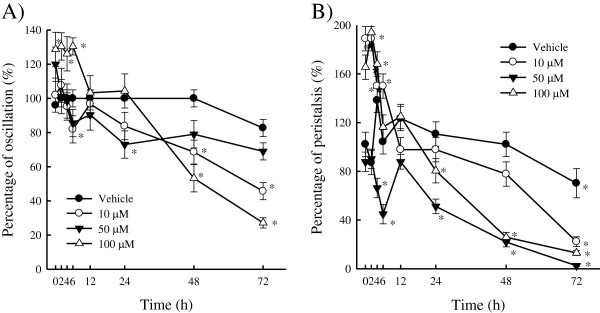
**Effect of yakuchinone A on *H. nana*.** Treatment with various concentrations of yakuchinone A (10, 50, and 100 μM) with incubation times of 2, 4, 6, 12, 24, 48, and 72 h on *H. nana*, respectively. Time course of effect on oscillation **A)** and peristalsis **B)** of *H. nana* of yakuchinone A presented as percentages. Vehicle is 0.1% DMSO solvent. Each value is presented as mean ± SD of three individual experiments; *p < 0.05 indicates a significant difference from the result for vehicle-treated worms.

Figure 6 plots the time course of oscillation and peristalsis during yakuchinone A treatment. In oscillation activity assay, the percentage of oscillation for the vehicle control (0.1% DMSO) decreased by about 18% from 72 h cultivation (Figure 6A). However, in the peristalsis activity assay, the percentage of peristalsis for the vehicle control (0.1% DMSO) decreased by 31% from 72 h cultivation (Figure 6B). The change of peristalsis of *H. nana* was more sensitive than that of oscillation via treatment of vehicle. Treatment with 10, 50, and 100 μM yakuchinone A has a greater effect on peristalsis than oscillation for 24, 48, and 72 h. Peristalsis activity disappeared before oscillation activity was lost when *H. nana* was dead. In fact, *H. nana* has no peristalsis or oscillation effect when dead.

In the oscillation activity assay (Figure 6A), exposure to 100 μM yakuchinone A for 72 h caused the maximum effect of 27% of *H. nana*. Treatment with yakuchinone A (a concentration of 50, 100 μM but not 10 μM) for 48 and 72 h reduced the oscillation up to 21% and 31% or 47% and 73%, respectively. Yakuchinone A slowly reduced oscillation from 2 to 72 h but did not cause death. Yakuchinone A reduced the oscillation activity of *H. nana* in a time- and dose-dependent manner for 24 to 72 h (Figure 6A).

The effect of yakuchinone A over time of the peristalsis activity of *H. nana* was investigated (Figure 6B)*.* For peristalsis activity assay, a dose- and time-dependent effect for 24 to 72 h was also observed by treatment with yakuchinone A. Treatment for 48 h with 50 and 100 μM yakuchinone A stopped peristalsis in more than approximately 21 to 25% of worms. Yakuchinone A at 50 and 100 *μ*M slowly reduced peristalsis from 2 to 72 h. Treatment with 10 μM yakuchinone A for 72 h reduced peristalsis to 22% (Figure 6B). This effect on peristalsis is stronger than on oscillation activity. The above performances were the same for other concentrations of yakuchinone A in peristalsis activity.

### Nematocidal activity against *A. simplex*

**Figure 7:**
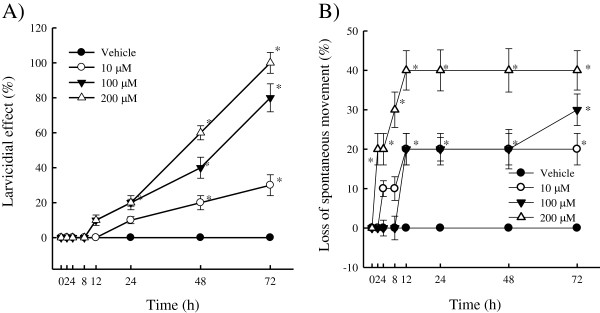
**Time course of larvicidal activity A) and loss of spontaneous movements B) of *A. simplex *by yakuchinone A treatment.** Effect of yakuchinone A (10, 100, and 200 μM) for 2, 4, 8, 12, 24, 48 and 72 h on third-stage larvae of *A. simplex*. Vehicle is 0.1% DMSO solvent. Each value is presented as mean ± SD of three individual experiments. Statistically significant, *p < 0.05 indicates a significant difference from the result for vehicle-treated worms.

In the first series of experiments, the larvicidal effects were used to study the ability of yakuchinone A to alter survival of AsL3. The time course of the yakuchinone A-induced loss of mobility on AsL3 was also studied. Figure 7A shows more than 20% of the worms had stopped moving at 72 h of treatment with 10, 100 and 200 μM yakuchinone A, whereas up to 10% of the larvae ceased movement activity at 12 h of treatment with 200 μM. Additionally, the maximum loss of spontaneous movement occurred at a concentration of 200 μM. Yakuchinone A caused a dose- and time-dependent loss of spontaneous movement. However, the vehicle (0.1% DMSO) had no effect on AsL3. Approximately, up to 20% of the larvae were dead at 48 h at 10, 100 and 200 μM yakuchinone A (Figure 7B), and up to 35% and 40% of the larvae were dead at 48 and 72 h, respectively, including 100 and 200 μM. Figure 7B shows *A. simplex* mortality was observed to be up 40% at 48 h after exposure to 100 and 200 μM yakuchinone A, which showed more lethal efficacy than against *H. nana* (Figure 6).

## Conclusions

Yakuchinone A isolated from *A*. *oxyphylla* scavenges radicals of biological interest and preventes damage to oxidative stress. The study suggests that yakuchinone A inhibits adipocyte differentiation in 3 T3-L1 cells. Treatment with yakuchinone A reduces the intracellular accumulation of neutral lipids and suppresses the induction of *leptin* and *PPAR*γ. Moreover, theses experimental results suggest that inhibition of cell growth by yakuchinone A is more significant for NMSC than for melanoma and noncancerous cells. Following incubation with yakuchinone A in BCC cells increases apoptotic body formation as well as down-regulated Bcl-2, up-regulated Bax, and increased cleavage PARP. Additionally, previous studies have shown that yakuchinone A has a stronger nematocidal activity of *A. simplex* than cestocidal activity of *H. nana.* These results support the development of selective and efficient natural anthelmintic compounds against helmineth or cestode (Additional file 1). Previous evidence has established that larvicide activity toward *A. simplex* does not depend on scavenging activity, and that free radicals can be harmful to *A. simplex*, for which the scavenging of these free radicals permits larvae to survive. However, this report is the first to verify that yakuchinone A has the cestocidal activity against *H. nana*, the scavenging activity against DPPH · and ABTS · ^+^ radicals, and the elimination effect on the spontaneous movement of AsL3. Therefore, the radical scavenging activity of yakuchinone A does not reduce its ability to stop the spontaneous movement of AsL3 or its cestocidal activity on *H. nana*. Further investigations must be conducted to elucidate the anthelmintic mechanisms of yakuchinone A against *A*. *simplex* and *H. nana* as well as its ability to eliminate the spontaneous movement of *A*. *simplex* and *H. nana* including their relationships to free radical scavenging activities.

## Abbreviations

AMPK: Adenosine 5′-phosphate-activated protein kinase; AP-1: Activator protein 1; DMSO: Dimethylsulfoxide; MDA: Malonaldehyde; NMSC: Non-melanoma skin cancer; ODC: Ornithine decarboxylase; PARP: Poly (ADP-ribose) polymerase; PPARγ: Peroxisome proliferator-activated receptor γ; ROS: Reactive oxygen species; TBARS: Thiobarbituric acid reactive substances; TNF-α: Tumor necrosis factor-α; TPA: 12-*O*-tetradecanoylphorbol-13-acetate.

## Competing interests

The authors declare that they have no competing interests.

## Authors’ contributions

RJL, LPC, HYD, CHL, acquisition of data; analysis and interpretation of data; statistical analysis; drafting of the manuscript; obtained funding; study supervision. All authors read and approved the final manuscript. CMY, FYC, administrative support; study supervision. THC, GHW: review of the manuscript. YPT, LW, TWH, HCW, acquisition of data.

## Pre-publication history

The pre-publication history for this paper can be accessed here:

http://www.biomedcentral.com/1472-6882/13/237/prepub

## Supplementary Material

Additional file 1 **Yakuchinone A exhibits antioxidative properties, anti-adipocyte differentiation, antitumor activity, and anthelmintic activities against *A. simplex *** **and *H. nana.***
Click here for file
