# Supplementary material for: Antioxidant, anti-adipocyte differentiation, antitumor activity and anthelmintic activities against Anisakis simplex and Hymenolepis nana of yakuchinone A from Alpinia oxyphylla
Source: BMC Complement Altern Med. 2013 Sep 26;13:237. doi: 10.1186/1472-6882-13-237 (PMC3879407; doi:10.1186/1472-6882-13-237)
Supplement: Additional file 1 — Yakuchinone A exhibits antioxidative properties, anti-adipocyte differentiation, antitumor activity, and anthelmintic activities against A. simplex and H. nana. [file 1472-6882-13-237-S1.pdf]

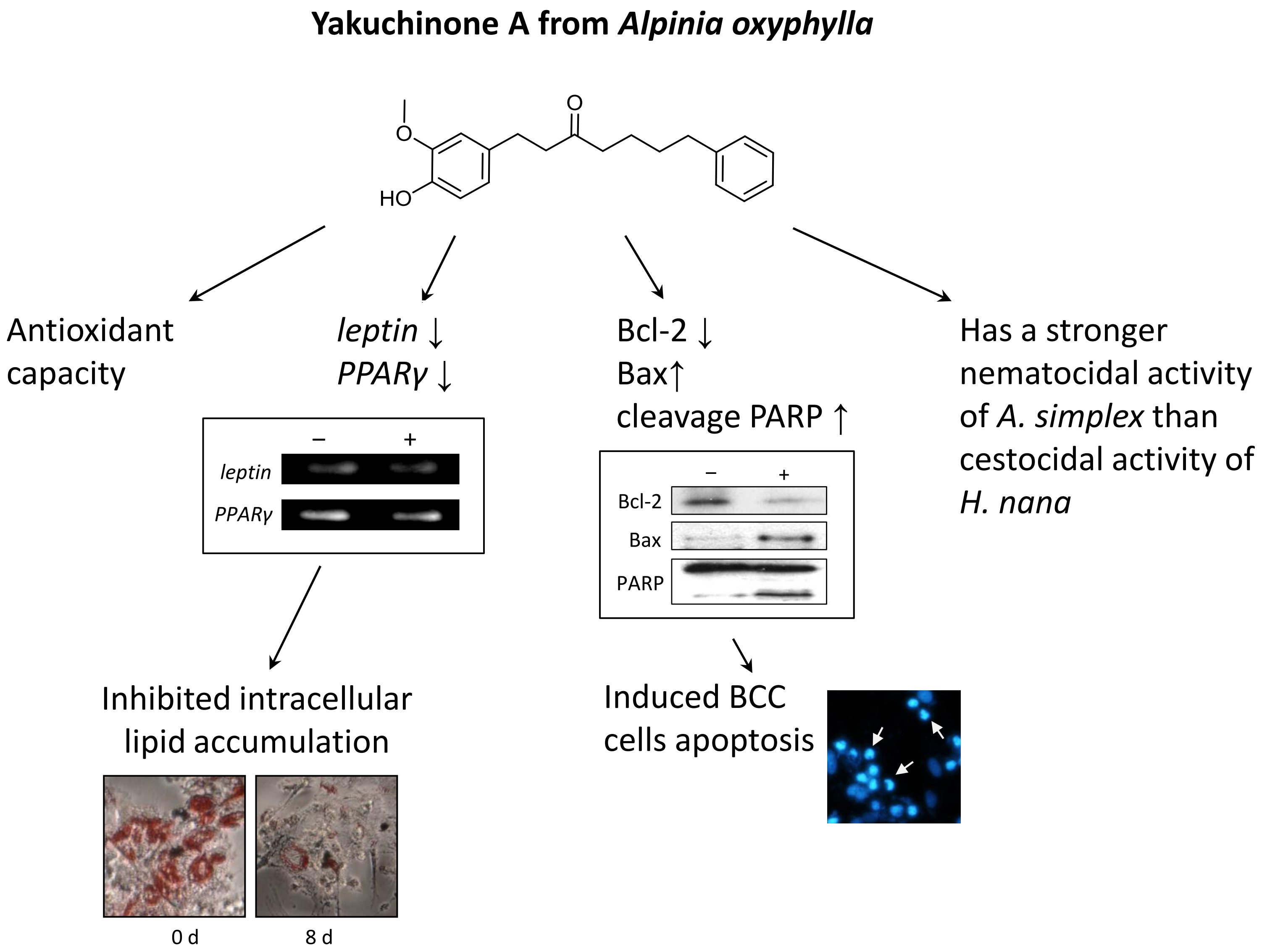
Yakuchinone A from *Alpinia oxyphylla*
O
HO
O
Antioxidant capacity
leptin ↓
PPARγ ↓
–
+
leptin
PPARγ
Inhibited intracellular lipid accumulation
0 d
8 d
Bcl-2 ↓
Bax↑
cleavage PARP ↑
–
+
Bcl-2
Bax
PARP
Induced BCC cells apoptosis
Has a stronger nematocidal activity of *A. simplex* than cestocidal activity of *H. nana*
